# The Impact of Pets on Everyday Life for Older Adults During the COVID-19 Pandemic

**DOI:** 10.3389/fpubh.2021.652610

**Published:** 2021-04-09

**Authors:** Jennifer W. Applebaum, Carlyn Ellison, Linda Struckmeyer, Barbara A. Zsembik, Shelby E. McDonald

**Affiliations:** ^1^Department of Sociology and Criminology & Law, University of Florida, Gainesville, FL, United States; ^2^Department of Occupational Therapy, University of Florida, Gainesville, FL, United States; ^3^School of Social Work, Virginia Commonwealth University, Richmond, VA, United States

**Keywords:** COVID-19, aging and public health, companion animals, human-animal interaction, pets, multispecies families, older adults, coronavirus

## Abstract

**Purpose:** The social and behavioral health of older adults is of particular concern during the COVID-19 pandemic. It is estimated that at least 50% of older adults in the U.S. have pets; while pets may be a source of support, they could also pose unique challenges during an already trying time. We aimed to investigate how pets impacted the everyday lives of older adults in the early stages of the COVID-19 pandemic.

**Methods:** A large survey of U.S. pet owners (*n* = 2,068) was administered to assess the impact of relationships with pets during COVID-19 on human health and well-being. We conducted bivariate analyses to compare levels of social support, loneliness, pet attachment, and family income for a subset of older adults (ages 65 and older) with a younger comparison group (ages 18–64). Using thematic and content analysis, we analyzed two open-ended prompts from age 65+ respondents (*n* = 122): (1) the pros and cons of living with pets during the pandemic, and (2) advice for those living with pets in future pandemics.

**Results:** Older adults, on average, reported lower levels of social support and less loneliness than respondents below age 65. There were no significant differences in strength of attachment to pets nor income between the younger and older respondents. For the open-ended prompt regarding pros and cons, we coded three emerging themes and related sub-themes: (1) pros (company; more time together; life purpose or meaning; love; support; stress relief; routine; distraction; exercise), (2) cons (general worry; potential for illness; limited participation; veterinary care access; obtaining supplies; difficulty meeting pet needs; financial concerns), and (3) no difference. Advice shared was coded into 13 themes/sub-themes: pets' health and welfare; make plans; veterinary information; treat pets like family; don't abandon pets; human health and well-being; stay calm; enjoy pets; keep routine; be careful of transmission; seek community resources; keep supplies stocked; and finances.

**Conclusions:** Pets may fulfill some social and emotional needs for older adults during this particularly isolating event; equally important to consider are the challenges that may be precipitated by and/or exacerbated by this public health emergency.

## Introduction

On January 30, 2020, the World Health Organization declared the recent SARS-CoV-2 outbreak (hereafter referred to as “COVID-19”) a global public health emergency. In March 2020, stay-at-home orders were put into place in the United States to “flatten the curve” and slow the spread of the virus. As a result of measures to prevent the spread of the virus, as well as the magnitude of mortality in the U.S. and globally, the COVID-19 pandemic has impacted our lives in many different ways. Social and behavioral health impacts may be greater for older adults (65 years and older) as they are at a greater risk of hospitalization or death if diagnosed with the virus (1). With stay-at-home orders and warnings to social distance, older adults may have limited their participation in meaningful life activities causing psychosocial strain (2). Therefore, older adults may have been spending more time indoors with a pet (i.e., companion animal^1^).

Many older adults in the United States share their lives and homes with pets. Recent estimates show that the prevalence of pet ownership tends to peak in mid-life: nearly 70% of Americans age 50–59 are estimated to have pets. Rates of pet ownership decline slightly with age to under 60% for 60–69 year-olds, and below 50% for those 70 and older (3). Despite the overwhelming popularity of pet ownership, relationships with pets vary widely in the U.S.; however, the majority of pet owners consider their pets to be family members and share strong attachment bonds with them [i.e., the “multispecies family”; (4)]. Previous research is mixed in terms of the effects of pet ownership on human health and well-being: in certain circumstances pets likely offer stress relief and companionship, while in others they may become a caregiving burden (5–8). It is important to conceptually separate the effects of *pet ownership* versus those of *positive relationships with pets* (sometimes referred to as the “human-animal bond”). *Pet ownership* tends to miss a great deal of nuance in human-animal relationships. In other words, the mere presence of a pet does not necessarily mean the relationship is mutually beneficial. Therefore, *positive relationships with pets* tends to better isolate the implied mechanisms that bestow benefits to pet owners. For example, the presence of a pet is not consistently found to benefit owners in terms of psychological health (8); however, there is increasing evidence that positive relationships with pets may buffer the deleterious psychological effects of stressful events (9–12).

Older adults may experience unique benefits and hardships associated with pet ownership as the natural aging process encompasses a variety of physical, cognitive, and social changes. Notably, falls, a leading cause of injury among older adults in the home, are linked to declines in various physical functions (13), and pet ownership may increase their potential (14). The changing needs across older adults' lifespan may impact an individual's ability to participate in meaningful activities of daily living (ADL) and instrumental activities of daily living (IADL) such as caring for a pet (6, 15). Older adults are also considered to be a vulnerable population, which could also impact their ability to care for a pet, as they experience health disparities or a higher burden of health conditions associated with social, economic, and/or environmental factors (16). Responsibilities associated with pets may cause additional stress during COVID-19, such as the disruption to routine, limited participation, and increased worry of meeting the pet's needs. For example, owning a pet during the pandemic may be particularly challenging for older adults as it may mean risking exposure to obtain their pet's supplies or care. Further, as the economic consequences of the pandemic progress, older adults who are aging-in-place with pets may be particularly vulnerable to housing insecurity (17–20), as pets are often restricted from affordable rental housing (21).

Due to the uniquely isolating experience of social distancing to prevent the spread of COVID-19, pets may provide specific benefits to older adults (22). Emerging research has shown that, overall, older adults experienced an increase in loneliness early on in the pandemic, with rates of loneliness improving over time (23, 24). While modern technology can afford individuals the ability to stay safely connected to one another during physical distancing, physically embracing someone outside one's household has been strongly cautioned against. In the absence of physical contact from other people due to social distancing measures, particularly for those living alone, pets can fulfill tactile needs and provide comfort via hugging, petting, or stroking (25). Relatedly, pets may have a buffering effect on loneliness in older adults, providing companionship and emotional support in the absence of human support, particularly in the context of a strong bond between the owner and pet (5). It is important to note, however, that recent research suggests strong attachment bonds with pets may be indicative of greater psychological vulnerability and lower resilience, particularly during adverse scenarios like the COVID-19 pandemic (26), and when social support from people may be lacking (27). Further, strong bonds with pets and low levels of social support have also been shown to predict delays in seeking healthcare (28, 29).

### The Current Study

At the beginning of the spread of COVID-19 in the U.S., a letter to the editor of the Journal of Gerontological Social Work predicted that pets would be both a resource for social support and companionship, but also a unique stressor for older adults (30). Indeed, emerging research suggests that their predictions were likely correct (26, 28, 31, 32); however, these predictions have not yet been tested in a sample specific to older adults, incorporating both quantitative and qualitative data to reveal, in their own words, how older adults perceived their relationships with pets during the current pandemic.

The One Health framework asserts that the health of people, animals, and the environment are interdependent (33). Included in the One Health model are human-animal interaction and the human-animal bond, which includes relationships between people and their companion animals (34, 35). In this study we take an overarching approach from the One Health framework toward understanding the ways that relationships with pets, and the responsibility of caring for a pet, impacts the health and well-being of older adults during the COVID-19 pandemic. In revealing patterns of positive, neutral, and negative experiences during this uniquely difficult and isolating time, we can gain insight into how to support human-animal relationships both during future hardships, as well as in the context of normal life.

In this study, we first identify group differences in attachment to pets, social support, loneliness, and income by comparing the younger subset (ages 18–64) to the subset of older adults (ages 65+). Next, we uncover themes related to living with pets during COVID-19 as reported in written responses by the subset of older adults in order to explain and elaborate upon results from the quantitative analysis. Data analyzed were collected in April through July of 2020 and capture the early effects of the pandemic on older adults' relationships with their companion animals, and the subsequent effects of those relationships on older adults' everyday lives.

## Methods

### Data

An anonymous survey was distributed on the Internet using Qualtrics survey software. 3,006 total responses were collected from April 6 through July 21, 2020. Inclusion criteria for eligible respondents included being age 18 and over and currently living in the United States with at least one pet/companion animal. The survey took approximately 30 min to complete and was available in English only. Topics included closed-ended and open-ended questions pertaining to interactions with pets, as well as social, economic, and demographic background information, and several questions related to health and well-being during the COVID-19 pandemic. Respondents also completed three validated scales included in the current study: the Lexington Attachment to Pets Scale (36), the Multidimensional Scale of Perceived Social Support (37), and the Three-Item Loneliness Scale (38). The screening questions and informed consent were mandatory response items; all subsequent survey questions were optional and could be skipped. A total of 2,068 respondents who responded to a question asking them to identify their age were included in the analyses in this study; 122 older adult respondents were included in the qualitative analyses. Respondents with missing information on the age variable were excluded.

### Compliance With Ethical Standards

This study was approved by the University of Florida's Institutional Review Board: protocol # IRB202000819. The researchers obtained informed consent from each participant, and participation was voluntary. Respondents were not compensated. Privacy of all participants' information was maintained according to University of Florida procedures.

### Participant Recruitment

Participants were recruited through snowball sampling. Recruitment advertisements were distributed to companion animal-related groups and accounts on Twitter, Facebook, Instagram, Reddit, and academic and professional special interest listservs, resulting in a convenience sample. The strengths and limitations of this recruitment method are discussed in the Limitations section below.

### Measures

#### Quantitative Measures

*Age*. Respondents were asked to report their age, in years. Ages ranged from 18 to 85^2^. For the purpose of this study, a binary variable was created in order to compare older adults (65+, coded 1) to the rest of the sample (coded 0). We defined “older adults” as individuals 65 years of age or older based on the increased risk of hospitalization and death for this age group (1), as well as the precedent set by academic studies of this population (39, 40).

*Social support*. Respondents completed the Multidimensional Scale of Perceived Social Support (37) to assess their perception of support from their social network. Respondents indicated their level of agreement on a five-point Likert scale to twelve statements such as, “There is a special person with whom I can share my joys and sorrows,” and “I can count on my friends when things go wrong.” Potential scores on this summated scale ranged from 12 (low social support) to 60 (high social support), *a* = 0.94.

*Loneliness*. Respondents completed the Three-Item Loneliness Scale (38) to assess the extent of loneliness they experienced both before the start of the COVID-19 pandemic, as well as at the time of data collection. Respondents were asked a series of three questions: “First, how often do you feel that you lack companionship?” “How often do you feel left out?” and “How often do you feel isolated from others?” Response options were on a three-point ordinal scale: “Hardly ever” (coded 1), “Some of the time” (coded 2), or “Often” (coded 3). Potential scores on this scale ranged from 3 (low loneliness) to 9 (high loneliness). Cronbach's alpha for scores prior to COVID-19 was 0.83; for during COVID-19 was 0.75.

*Attachment to pets*. Respondents completed the Lexington Attachment to Pets Scale (36), a 23-item measure of individuals' emotional attachment to their companion animal(s). Respondents indicated their level of agreement on a four-point Likert scale to statements such as, “Quite often I confide in my pet,” and “I believe my pet is my best friend.” Potential scores on this summated scale ranged from 32 (low pet attachment) to 92 (high pet attachment), *a* = 0.90.

*Income*. Yearly family income was reported in 26 groups that ranged from “ <$1,000” to “$170,000 or higher.”

*Gender*. Respondents reported their gender as man, woman, or other, which included anyone who selected categories for both man and woman, and/or those who selected a category for genderqueer/gender non-conforming.

*Education*. Level of education was reported by the respondent in categories: less than high school; high school or equivalent; some college; two-year college degree; four-year college degree; and graduate degree.

*Race/ethnicity*. Respondents reported their race and ethnicity in categories: non-Latinx White; non-Latinx Black, non-Latinx other race, non-Latinx multiracial, and Latinx.

#### Qualitative Measures

In addition to the quantitative measures, participants were asked to respond to open-ended questions. Two open-ended questions were used for this analysis. The first prompt asked, “what are the pros and cons of living with pets during coronavirus/COVID-19?” The second prompt asked, “is there any advice you would give to other people living with pets in future pandemics?”

### Data Analysis

#### Quantitative Procedures

Bivariate associations (*t*-tests) were used to assess differences in social support, loneliness, pet attachment, and income between older adults (age 65+) and the younger comparison group (ages 18–64). Listwise deletion was used to account for any missing observations on variables of interest, therefore sample size varies across each set of analyses. All quantitative analyses were conducted with Stata version 15.1.

#### Qualitative Procedures

Three research team members independently coded the data for two open-ended questions. Triangulation, the use of multiple coders, assured reliability and guided the development of a comprehensive understanding of phenomena building on multiple perspectives (41). Each research member provided expertise and perspective from their field including human-animal interaction, public health, and occupational therapy. Researchers utilized Microsoft Excel software to manage data for thematic and content analysis. The development of the codebook was based on quantitative measures of interest and emerging themes from a preliminary round of coding. After the first round of coding, the codebook was revised after team input and the second cycle of coding produced salient themes. We analyzed intercoder agreement, or the percentage of agreement among coders, for the first 30% of the data to ensure consensus and promote reflexivity (42). When discrepancies arose between coders, the final counts for themes and sub-themes were determined by the first author.

## Results

### Sample Characteristics

Select sociodemographic characteristics of the entire study sample (n = 2,068) are reported in this section. The majority of respondents (89.5%) identified as women; the remainder identified as men (7.8%), or “other,” which included non-binary, genderqueer or gender non-conforming, or a different identity (2.8%). Most respondents identified as non-Latinx White (87.5%); under one percent were non-Latinx Black (0.9%), 5.1% were non-Latinx other race, 1.9% were non-Latinx multiracial, and 4.7% identified as Latinx/Latino/Latina. Respondents reported their level of education: 42.9% had earned a graduate degree, 33.3% had a four-year college degree, 7.9% had a two-year college degree, 11.8% had attended some college but did not earn a degree, 3.6% had a high-school diploma or equivalent (i.e., GED), and 0.4% reported an educational level below high school graduation. Additional descriptive information is presented in Table 1 below.

**Table 1:** Descriptive information and bivariate associations displaying age group differences for all variables of interest.

**Variable**	***M* or % (*n*): full sample**	***M* or % (*n*): 65+ subset**	***M* or % (*n*): 18-64 subset**	**Range: full sample**	***p*-value**
Age	39.6 (2,068)	69.5 (122)	37.8 (1,946)	18-85	-
Social support score	48.5 (2,035)	44.4 (120)	48.8 (1,915)	12-60	0.000
Loneliness before COVID-19	5.1 (2,060)	4.5 (120)	5.1 (1,940)	3-9	0.000
Loneliness during COVID-19	5.6 (1,774)	5.1 (97)	5.6 (1,677)	3-9	0.011
Pet attachment score	81.1 (1,695)	80.4 (94)	81.2 (1,601)	32-92	0.418
Income group	$60,000-74,999 (1,909)	$60,000-74,999 (105)	$60,000-74,999 (1,804)	< $1,000 - $170,000+	0.234

*M = mean, p-values displayed from t-test results for age group differences*.

### Quantitative Results

In order to assess comparability of the younger group (18–64) and the older adult group (65+), we used chi-squared tests investigate any significant differences by gender, education, income, and race/ethnicity. No significant differences were found, therefore we consider the groups to be comparable by these select sociodemographic characteristics.

Table 1 contains descriptive information for all variables included in the analyses, as well as bivariate analyses for age group differences in attachment to pets, social support, loneliness, and income. The average age of the entire sample (*n* = 2,068) was 39.6 (S.D. = 13.7); among 122 older adults, aged 65-85, the mean age was 69.5 (S.D. = 3.9), and among younger respondents, aged 18-64, mean age was 37.8 (S.D. = 11.8). Strength of attachment to one's pet did not differ significantly between younger and older subsets (*t*(1,693) = 0.81). Subjective assessment of social support differed significantly by age group: older adults (65+) reported lower levels of social support than their younger counterparts [*t*(2,033) = 4.67]. Older adults also reported significantly lower levels of loneliness both prior to [*t*(2,058) = 3.81] and during the COVID-19 pandemic [*t*(1,772) = 2.56]. There were no significant differences in income between the younger and older groups [*t*(1,907) = 1.17].

### Qualitative Results

The researchers analyzed a total of 222 responses from the older adult subset (*n* = 122 older adults): 117 regarding the pros and cons of living with a pet during the pandemic and 105 soliciting participants' advice for others living with pets during future pandemics. Codes were not mutually exclusive as multiple themes and sub-themes could be relevant to each response (e.g., a participant's response could contain both pros and cons). Frequency counts reflected the number of participants that identified one of the themes or sub-themes within their response (e.g., a participant that mentioned multiple pros was only counted once). The overall intercoder agreement among the first 30% of responses for both open-ended responses was 98.21%.

#### Pros

A total of 94 participants (80.34%) discussed the pros of living with pets during the pandemic, dominating the responses. Sometimes participants explicitly identified topics as a pro and other times it was implied. The topics primarily associated with pro included company, more time together, distraction, providing life meaning/purpose, love, support, stress relief, routine, and exercise.

Company (also referred to as companionship) was discussed by 48 participants (41.03%), making it the most discussed topic of the pros identified. Participants emphasized that their pets were “excellent company” and due to the pandemic, pets “keep [participants] company because [participants are] home more.” Thus, the pro of having more time together with their pets is interconnected with companionship. For example, one participant shared that their pet “is a wonderful companion so the pro is that it is enjoyable to be home and be able to spend time with her.”

The second most discussed topic was that pets could act as a distraction for older adults. As a result, the participants were able to “focus on something fun.” Participants shared that pets “are not worried about the virus so they are always happy.” However, their distraction was not always considered a net positive, as one participant shared that “sometimes [their pet's] bid for attention can get in [the] way of work and makes [them] stop to connect.”

Older adults also reported pets' ability to provide support during the pandemic. Participants explicitly shared how their pet supported them emotionally (e.g., “the pro is the emotional support and entertainment they provide”) and others implicitly (e.g., “pros are that pets provide comfort while their owners are stuck at home”). One participant even shared that they “need to touch a living being,” demonstrating how their pet has physically provided comfort. Along with support, pets were a form of stress relief for older adults because pets could act as a “mood elevator.” One participant shared that they “have no idea how [they] would cope with the stress [if they] were without pets.”

Older adults discussed two pros at the same frequency: (1) how pets could provide love (e.g., “the love [their pets] give me”) and (2) how living with pets could bring a sense of life meaning or purpose. Participants reported their pets provided “unconditional love” and feeling “more purposeful” as their pet “gives another dimension to [their] life.” Participants highlighted another pro regarding the routine involved caring for a pet and how the routine aided older adults' desire for a “sense of normalcy.” As one participant explained, “being needed helps me feel normal… I have structure in my day based on my dogs needs for walks and play.” Additionally, three participants indicated pets can contribute to exercise, providing “an excuse for fresh air and walks.” Overall, older adults shared more pros than cons, and their responses demonstrated the multifaceted benefits of living with pets during COVID-19.

#### Cons

Following the discussion of pros, cons were mentioned by 32 participants (27.35%). Topics associated with cons included general worry, limitations in participation, access to veterinary care, difficulty obtaining supplies, and financial concerns. Further sub-themes explored older adults' worries of becoming sick, separation from their pet, and their ability to meet the needs of their pet.

A total of 14 older adults (11.97%) most frequently discussed the difficulties faced obtaining supplies for their pets. One participant noted that “it's harder to find the food and treats [their pets] like, and for some reason, it's harder to get kitty litter.” Participants indicated that they were “concerned about supplies” for their pets as “hoarding occurs making food and supplies scarce.” Considering participant's concerns with exposure, it may be “… harder to obtain supplies, unless you do delivery.”

Living with pets during the pandemic appeared to increase participants' general worries. If not just because there are “more lives to worry about,” participants shared specific worries potentially increased by the pandemic. Some participants discussed their worries of becoming sick and, specifically, “what would happen to [their] pets if [the participant] end[ed] up in the hospital.” Related to becoming sick, participants voiced worries regarding being separated from their pet and the stress it may cause their pet (e.g., “I had to be hospitalized for 4 days a couple of years ago and the mutts were very disturbed by the situation”). Participants also indicated it could be difficult meeting the needs of their pets which could also add to their worries. For example, one participant shared that their pets “can become demanding for treats throughout the day.” In general, a small group of participants revealed that living with pets during the pandemic could add to their daily worry and stresses associated with their care.

Further cons consisted of access to veterinary care, the limitations of participation in everyday activities, and financial concerns. A total of six participants (5.13%) shared that access to veterinary care was impacted during the pandemic as it could be “hard[er] to see [a] vet.” The pandemic certainly limits participation in everyday activities, and this is true for older pet owners as well. Participants shared that the pandemic made it “harder to participate in group pet activities (like dog parks or competitions)” and that their pets “can't visit [their] friends.” Financial concerns were mentioned the least for the identified cons at only two times (1.71%). One participant shared that if they were to become sick “vet care and funds are limited.” Another participant shared that there can be “money stress if you have lost your job.” Thus, older adults believe there are some disadvantages to living with their pets during the pandemic that could impact both human and animal health and well-being.

#### No Difference

A total of nine participants (7.69%) indicated that there was no difference in living with pets prior to the pandemic. For example, one participant shared that they “talk to [their pets] all day…but [they] did that before the coronavirus.” Table 2 provides a complete overview of all the themes and sub-themes for responses to the pros and cons prompt.

**Table 2:** Pros and cons with living with pets themes and sub-themes with counts.

**Theme**	**Sub-themes**	**Count**	**Example quotations**
Pros	1 Company	48	“I cannot imagine how lonely I would be especially now, if I did not have them.”
	1.2 More time together	8	“Out of school grandchildren have more time to play (multigenerational household). Pets getting more time with all family members.”
	2 Distraction	18	“[Pets] keep your mind from obsessing over uncontrollable things.”
	3 Support	15	“[Their] pets are a great comfort to me during this pandemic.”
	3.1 Stress Relief	9	“Family [is] receiving more companionship and stress relief from pets.”
	4 Love	8	“There are only pros – LOVE.”
	5 Life meaning/purpose	8	“As always it gives a sense of purpose caring for another living thing.”
	6 Routine	5	“[Pets] are calming and add to the feeling of normalcy when I'm home.”
	7 Exercise	3	“[Pets] keep me active”
Cons	1 Difficulty obtaining supplies	14	“Having to go out to buy dog food is a con.”
	2 General Worry	3	“Con is more lives to worry about.”
	2.1 Worry of becoming sick	8	“The only con is worrying what would happen to him if I die. Or go to hospital. How he would not understand. I have always prayed I outlive him, so he doesn't suffer.”
	2.2 Separation	2	“I worry about them possibly catching it, about being separated from their company if I catch it.”
	2.3 Meeting needs of pets	4	“Cons are they can become demanding for treats throughout the day.”
	3 Access to vet care	6	“Vet care is urgent care only.”
	4 Limits participation	3	“Harder to participate in group pet activities (like dog parks or competitions).”
	5 Financial concerns	2	“Money stress if you have lost your job.”
No difference		9	“For me, the pros and cons are the same as before because I am retired.”

*Themes and sub-themes were not mutually exclusive*.

#### Advice

The advice given by pet owners provides another opportunity to explore the impact of relationships with companion animals during the pandemic. Advice focused on both pets' and humans' health and welfare/well-being with other topics including ensuring supplies are stocked, being careful of transmission, keeping a routine, seeking community resources, and securing finances. The largest sub-theme for advice given was comprised of responses from 29 participants (27.62%) who expressed the importance of having supplies stocked, mentioning items such as food and medicine.

A total of 21 participants (20%) discussed pets' health and welfare. Generally, participants shared that individuals should “keep pets clean and healthy” reminding people that pets “depend on you.” Participants also emphasized the importance of making plans, especially “in the event you get sick.” Making plans also involves having crucial veterinarian information which could change during the pandemic (e.g., hours open and associated policies). The advice also focused on treating pets like family members, as many older adults view their pets as family, and not abandoning pets.

Participants emphasized the importance of taking care of oneself and also share how pets can increase their health and well-being. For example, one participant shared that pets:

lower your stress and blood pressure. Dogs will keep you healthy by going on walks, but any [pet] will give you much more than you give them. Hold onto your pet! He or she may be your last best friend.

Human health and well-being advice also focused on “stay[ing] calm” and taking the time to enjoy “spending more time with [pets].”

Furthermore, eight participants' (7.62%) advice centered on being careful of transmission of the virus for both the owner and the pet while three other participants pointed out the need to keep a routine. Participants offered advice to those living in future pandemics with pets to “not be afraid to reach out” to community resources for assistance with “vet bills” and food. Additionally, older adults affirmed the need to secure finances by even “prepar[ing] financially for their [pets] care in [their] will.” Additional quotations and counts are provided in Table 3.

**Table 3:** Advice for living with pets during future pandemic themes and sub-themes with counts.

**Themes and sub-themes**	**Count**	**Example quotations**
1 Supplies stocked	29	“Always be prepared with enough food and supplies to care for any animals you are responsible for.”
2 Pets' health and well-being	21	“Keep pets clean and healthy. They depend on you.”
2.1 Make plans	18	“Make sure you have a plan in place in the event you get sick for your pets.”
2.1.1 Veterinarian information	5	“…know your veterinarian's policies during pandemics.”
2.2 Treat pets like family members	9	“I would treat them the same as a human family member if they got sick.”
2.3 Do not abandon pets	7	“Do not give your pet up unless you absolutely need to. Your pet would rather stay with you and share your illness than sit in a shelter not knowing why they were sent away.”
3 Human health and wellbeing	20	“The emotional support from pets make them invaluable during something as stressful as a long-term pandemic.”
3.1 Stay calm	4	“Just stay calm and love your pets.”
3.2 Enjoy	16	“Have fun with your pet…it will help you both.”
4 Be careful of transmission	8	“Wash your hands, wear the mask and gloves. Keep yourself healthy. If not for your sake then do it for them. They depend on you.”
5 Keep routine	3	“Keep to normal routine as much as possible.”
6 Seek community resources	2	“Not to be afraid to reach out if you are in need of food–there are a couple food banks in my community of which I support…don't risk losing your pet.”
7 Finances	2	“Set aside money for their care.”

*Themes and sub-themes were not mutually exclusive*.

## Discussion

In this study, we investigated how pets impacted the everyday lives of older adults during the early stages of the COVID-19 pandemic in the United States. We first explored differences between older adults (65+) and a younger comparison group (18–64 year-olds) in social support, loneliness, attachment to pets, and income. Next, we analyzed written responses to open-ended prompts from our subset of older adult respondents to expound and compare to quantitative results. We found that, compared to their younger counterparts, older adults reported lower levels of social support, and conversely, lower levels of loneliness both before the start of the COVID-19 pandemic, as well as during. In general, social support may decrease with age (43), and poor support among older adults is known to be associated with poorer health status, compared to those with adequate support (44). Our findings were consistent with recent, COVID-19-related research showing lower levels of loneliness in older adults, compared to their younger counterparts (23); one study of older adults suggested that loneliness, and subsequent sleep problems, were attenuated via resilience (45). Given our qualitative findings showing that the older adults in this sample overwhelmingly found their pets to offer companionship and support, it is possible that their pets may have played a role in their resilience, therefore helping owners to feel less lonely, both before and during the pandemic. This is also consistent with findings in a U.K. sample from Ratschen and colleagues (26), which suggested that pet ownership may offer some moderation of loneliness during the pandemic. Indeed, previous research has shown that older adults often cite companionship as the main reason for owning a pet (6). Also reflected in qualitative findings were concerns related to a lack of instrumental social support (i.e., tangible help provided by others), such as contingency care plans for pets if the respondent were hospitalized or incapacitated from a severe case of COVID-19. It is also noteworthy that responses from older adults regarding “cons” of living with pets during the pandemic were generally in the realm of challenges related to pet ownership during this time, rather than downsides. Taken together, this suggests that pets may help provide *emotional* social support and could be a positive physical presence offering tactile comfort (25) that mitigates loneliness, but they are unable to offer the same types of multidimensional social support as people (i.e., instrumental support).

Older adults did not differ from the younger group in their strength of attachment to their pet; pet attachment was relatively high in the entire sample, as was expected given the salience of the study topic to those interested in pets. We found that strong pet attachment, as well as general positive attitudes toward companion animals, was often implied in the advice given by older adults for pet owners in future pandemics. These responses also reflected the One Health concept of interconnected human and animal health (33). For example, older adults often discussed how their pets' health and welfare was important to prioritize, while also implicating pets in the maintenance of their own health and well-being. Also directly relevant to the One Health framework were concerns about zoonotic disease management of COVID-19, in terms of keeping oneself and ones' pets safe, and preventing intra-household disease spread between people and pets in multispecies families. Future research might consider the impact of attachment to pets on the management of zoonotic disease transmission when both companion animals and humans are susceptible.

Considering the unique issues that economically insecure older adults with pets face, such as securing pet-friendly housing (17–20), and accessing veterinary care (46), we were interested in whether the older adults in our sample may be more economically vulnerable than the younger comparison group of respondents. We did not find differences in income between older adults and the younger group in our sample. Overall, our sample reported relatively high income, as compared to the U.S. population. As the economic fallout of the COVID-19 pandemic was unknown when these data were collected and our sample tended to be economically secure, it is unlikely our respondents were overwhelmingly concerned about finances. This was reflected in qualitative responses indicating that respondents relied on supply delivery in order to avoid disease exposure, which can be cost-prohibitive. However, a few older adults did mention vague concerns related to money in terms of affording veterinary care, as well as a general awareness of potential impending economic insecurity. This was demonstrated in recommendations made to seek out community resources if experiencing financial hardship related to the pandemic and preparing financially in general. It is possible our findings would be different if these data were collected later into the pandemic, as inequalities were further exacerbated in the U.S. (47). Future research should investigate how these issues may be different for lower-income older adults.

Older adults also expressed worries related to caring for pets during the pandemic, such as issues with safely obtaining supplies, accessing veterinary care, and planning for contingency pet care if they were to become sick. Participants also mentioned concerns related to meeting the social and behavioral needs of pets while also mitigating the risk of infection. As is reflected in recent research from the U.K. and Spain, while individuals were spending more time overall with their pets, they were also finding it difficult to exercise and socialize them (32, 48). While there is a particular concern for the welfare of pets and the emergence of new behavioral issues (e.g., separation anxiety) when people go back to regular work outside the home, older adults may be an exception. For example, some of our respondents mentioned that they did not experience any differences in life with their pet(s) during the pandemic as compared to before, as many were presumably retired and potentially spent a great deal of time at home with their pet already. It is also important to note that older adults may continue to be involved in the community after retirement through activities (e.g., volunteering and employment) that may also have been suspended due to COVID-19 (49).

Additional qualitative findings included the lack of discussion on related exercise, and how living with pets gave older adults life meaning or a sense of purpose. Research examining the effects of pet ownership among older adults has focused on physical activity [e.g., physical health outcomes associated with dog walking; (5)]. Dog walking may be a way to combat age-related declines in physical activity (50), yet participants only discussed exercise three times. Perhaps our sample's high average socioeconomic status was related to their ability to complete physical activity in other ways (e.g., paid membership to a gym) and also afforded them the option of paying for dog walkers. It is also possible that the infrequent mention of exercise in our sample was a result of pet type; for example, cats do not require outdoor walks with their owners. Participants also shared that pets could be a source of life meaning or purpose, which is strongly associated with positive health outcomes among older adults (51). Indeed, previous research suggests that taking care of pets gives older adults a sense of responsibility and purpose in completing various tasks to ensure their pets' care [e.g., preparing meals and keeping a routine; (52)].

Several respondents specifically voiced their concern about pet abandonment in responses to our open-ended prompt asking for advice for pet owners during a future pandemic. Indeed, there is growing concern that the increased popularity of pets during the pandemic combined with the continued economic downturn will result in a massive increase in abandoned and shelter-relinquished pets (31). It is yet to be seen if these fears will manifest, but tens of millions of Americans are facing eviction in 2021 (53), which will likely result in many families being forced to give up their pets. The resulting implications for both human well-being and animal welfare, and dog and cat euthanasia rates, could be substantial.

### Implications

Our findings suggest that pets may be an important source of support and normalcy for older adults during the COVID-19 pandemic and beyond, and most view them as family members. Our results also provide useful insights of potential challenges older pet owners may face in the event another pandemic or similar hardship occurs. The pandemic's disruption may have revealed more of the nuanced benefits (e.g., emotional support) and disadvantages (e.g., another stressor) of pet ownership among older adults. Findings suggest the pandemic has increased worry among older adults caring for pets and as a result, older adults with pets may benefit from special assistance during public health emergencies. For example, to mediate these concerns, families, friends, and communities may provide assistance with safely procuring pet supplies and food, support for pets with behavioral issues, or making arrangements for contingency care in the event of owner illness. We recommend incorporating consideration of pets into family social services, particularly for economically vulnerable older adults, with the goal of keeping multispecies families together through adversity.

### Strengths and Limitations

A major strength of this study was the responsiveness of the data collection period: to our knowledge, it is the only dataset to capture these measures of human-animal interaction in the U.S. in the very early stages of the COVID-19 pandemic. As a result of the rapid nature of data collection, our recruitment strategy was convenience-based and thus our findings cannot be generalized to the entire pet-owning population in the U.S. As is common with surveys pertaining to companion animals that are recruited via convenience and snowball sampling, our sample was made up primarily of non-Latinx White women who had high average family income and a high average level of education. While non-Latinx White individuals tend to have the highest rates of pet ownership in the U.S., compared to other races/ethnicities, rates of pet ownership do not vary much by gender or socioeconomic status (3). Probability-based sampling that enables results to be generalized to all pet owners at the U.S. population-level might reveal patterns not evident in this study sample, particularly issues related to a lack of resources or racial or ethnic discrimination, and is recommended for future research. Additionally, our sample was limited to a small subset of older adults (*n* = 122). While our results indicated no significant differences by selected sociodemographic characteristics, there are limitations related to comparing older adults with a broad range of ages (i.e., 18–64), as younger and middle adulthood encompass a wide variety of developmental stages and may lack some nuance that could impact results. Future research may consider the questions posed in this study from a life course perspective. Additionally, as this study used bivariate tests of association to compare groups, it should be noted that the differences between age groups did not account for potential confounders. Future research should employ multivariate analyses to isolate the effects of various respondent characteristics that may further explain variation in responses by age group.

## Conclusion

Taken together, our results show that pets played a unique role for older adults during the early stages of the COVID-19 pandemic in the United States. Pets were both a comfort and source of companionship and support, while also a source of stress and worry. Overall, consideration of both the benefits and detriments of relationships with pets among older adults is needed to support multispecies families during emergencies such as COVID-19.

## Data Availability Statement

The dataset for this study is available upon reasonable request from the first author.

## Ethics Statement

The studies involving human participants were reviewed and approved by University of Florida Institutional Review Board. The patients/participants provided their written informed consent to participate in this study.

## Author Contributions

JA and CE: conceptualization, methodology, and writing. JA, CE, and LS: formal analysis. LS, BZ, and SM: supervision and resources. All authors review and editing.

## Conflict of Interest

The authors declare that the research was conducted in the absence of any commercial or financial relationships that could be construed as a potential conflict of interest.

## Footnotes

^1^We use the terms “pet” and “companion animal” interchangeably throughout to refer to a domesticated animal that lives with and is taken care of by the respondent and/or a member of their household.

^2^We did not specifically limit the subset of older adults to 85 years of age. The oldest respondent was 85 years of age.
